# Three new species of *Hohenbuehelia* (*Agaricales*, *Basidiomycota*) from China based on morphological and multi-locus phylogenetic evidence

**DOI:** 10.3897/mycokeys.142.204939

**Published:** 2026-10-22

**Authors:** Ting Luo, Nian-Jie Che, Wei-Chao Feng, Yang-Yang Cui, Jing Li

**Affiliations:** 1 Faculty of Geography, Yunnan Normal University, Kunming, China Faculty of Geography, Yunnan Normal University Kunming China; 2 CAS Key Laboratory for Plant Diversity and Biogeography of East Asia, Kunming Institute of Botany, Chinese Academy of Sciences, Kunming, China CAS Key Laboratory for Plant Diversity and Biogeography of East Asia, Kunming Institute of Botany, Chinese Academy of Sciences Kunming China; 3 College of Life Sciences, University of Chinese Academy of Sciences, Beijing 100049, China College of Life Sciences, University of Chinese Academy of Sciences Beijing China; 4 Yunnan Key Laboratory for Fungal Diversity and Green Development, Kunming, China Yunnan Key Laboratory for Fungal Diversity and Green Development Kunming China

**Keywords:** Morphological characters, multigene sequence, novel species, taxonomy

## Abstract

The genus *Hohenbuehelia*, widely distributed in temperate and tropical regions, plays an important role in forest ecosystems. However, the species diversity of *Hohenbuehelia* in China has been underestimated. This study presents the morphological and molecular characterization of three novel *Hohenbuehelia* species from China, enhancing the understanding of this genus in the region. *Hohenbuehelia albomarginata* is characterized by small, gray-brown to brown basidiomata with a frosted, concolorous tomentum on the pileus, often white at the margin, and elongate basidiospores. *Hohenbuehelia striata* differs in having brown, glabrous basidiomata that are laterally or dorsally attached, lacking a stipe or pseudostipe, with a very faintly translucent-striate pileus margin and ellipsoid to broadly ellipsoid basidiospores. *Hohenbuehelia guizhouensis* has a gray-brown to brown pileus covered with white tomentum, lamellae radiating from the point of attachment, and elongate, ellipsoid to subreniform basidiospores. Phylogenetic analyses based on multigene sequence data further support their taxonomic distinctiveness. All species are comprehensively described, illustrated, and compared with closely related and morphologically similar taxa. This study expands the known diversity of *Hohenbuehelia* and highlights the need for further exploration of fungal diversity in China.

## Introduction

*Hohenbuehelia* was validated by Schulzer in 1866 and belongs to *Pleurotaceae* Kühner, *Agaricales* Underw (Kirk et al. 2001). It is a cosmopolitan genus and comprises about 202 valid species (http://www.indexfungorum.org), for more than 50 accepted species (Liu et al. 2010; Consiglio et al. 2018; He et al. 2019). It is widely acknowledged that a few *Hohenbuehelia* species, such as *H. reniformis* (G. Mey.) Singer and *H. petaloides* (Bull.) Schulzer, have been reported as edible mushrooms (Mao 1998). More importantly, several species are good sources of antioxidant properties as well as antitumor and antiviral activities (Shipley et al. 2006; Li et al. 2017; Wang et al. 2019). It is generally accepted that some species in this genus, attacking nematodes through production of adhesive knobs on its hyphae or on germinated asexual conidia, perform an important role in biological control of nematodes (Drechsler 1941; Barron 1977; Thorn and Barron 1984; Thorn et al. 2000; Li et al. 2014; Lubian et al. 2021). The diversity of bioactive compounds from *Hohenbuehelia* species and their potential applications underline the importance of detailed taxonomic study of this genus.

Species within *Hohenbuehelia* are mainly characterized by small basidiomata, a spathulate, flabelliform or half-circular pileus, lamellae that are decurrent or radiating from the point of attachment, a reduced or absent stipe, ellipsoid basidiospores, and thick-walled metuloid pleurocystidia (Singer and Digilio 1951; Thorn and Barron 1986; Corner 1994; Li et al. 2014). There has been a growing interest in taxonomic research of the genus, mostly in North and South America, Europe, Southeast Asia (Albertó et al. 1998; Courtecuisse et al. 1999; Silva-Filho and Cortez 2017; Consiglio et al. 2018; Holec and Zehnálek 2020; Mentrida 2016; Phonemany et al. 2023). As a pleurotoid fungus, *Hohenbuehelia* was historically placed in the genus *Pleurotus* (Fr.) P. Kumm. due to its similar pileus shape (Thorn and Barron 1984, 1986; Barron and Thorn 1987). However, phylogenetic analyses based on the internal transcribed spacer (ITS) region and large subunit ribosomal RNA gene (nrLSU), together with the presence of a gelatinous layer between the context and the cuticle, support the separation of *Hohenbuehelia* from *Pleurotus* and confirm its status as a monophyletic genus within *Pleurotaceae* (Thorn et al. 2000; Moncalvo et al. 2002). As more and more species are described in *Hohenbuehelia*, different researchers have inconsistent views on the infrageneric classification. Using morphological and molecular phylogenetic data, about fourteen taxa have been identified in China (He 1992; Liu and Bau 2009; Liu et al. 2010; Li et al. 2014; Qi et al. 2023; Xu et al. 2023). Despite this progress, the species diversity of *Hohenbuehelia* in China is still poorly understood. A broader taxon sampling coupled with both molecular and morphological data is needed to fully understand the species diversity in China.

Here, three new species of *Hohenbuehelia* from China, *H. albomarginata*, *H. guizhouensis* and *H. striata*, are described based on both molecular and morphological evidence. These species are generally distributed in subtropical coniferous and broad-leaved mixed forests and warm-temperate deciduous broad-leaved forests.

## Materials and methods

### Specimen collection

The nine specimens examined in this study were collected from subtropical broad-leaved forests in Yunnan, Guizhou, Sichuan and warm-temperate deciduous broad-leaved forests in Hebei, China between 2002 and 2024. All specimens are deposited in the Herbarium of Cryptogams, Kunming Institute of Botany, Chinese Academy of Sciences (KUN-HKAS).

### DNA extraction, PCR amplification and DNA sequencing

The genomic DNA was extracted from silica-gel dried or herbarium material using the CTAB method (Doyle and Doyle 1987). The universal primer pairs ITS1F/ITS4 (White et al. 1990), LR0R/LR5 (Vilgalys and Hester 1990), EF1-983F/EF1-1567R (Rehner and Buckley 2005) were used to amplify internal transcribed spacer (ITS) region, large subunit ribosomal RNA gene (nrLSU) and translation elongation factor 1-α (*TEF*1*α*), respectively. The PCR reaction was conducted on an SimpliAmp^TM^ Thermal Cycler (Applied Biosystems, Foster City, CA, USA) under the following conditions: 94 °C for 4 min, followed by 35 cycles of 94 °C for 40 s, 52 °C for 1 min, and 72 °C for 1 min; afterwards, a final extension at 72 °C for 8 min. The PCR products were purified using a Gel Extraction and PCR Purification Combo Kit (Spin-column; Bioteke Corporation, Beijing, China). The purified products were sent to Sangon Biotech (Shanghai, China) for sequencing on an ABI-3730-XL sequence analyzer (Applied Biosystems, Foster City, CA, USA) using the same primer combination as for the PCR.

### Phylogenetic analyses

Sequences newly generated and retrieved from GenBank were listed in Table 1. *Pleurotus ostreatus* (Jacq.) P. Kumm, *Pleurotus citrinopileatus* Singer, *Pleurotus djamor* (Rumph. Ex Fr.) and *Pleurotus tuoliensis* (C.J. Mou) M.R. Zhao & Jin X. Zhang were chosen as outgroups according to the recent phylogenetic studies by Consiglio et al. (2018) and Xu et al. (2023). The datasets were then aligned using MAFFT v7.130B (Katoh and Standley 2013) and manually optimized in BIOEDIT v7.0.9 (Hall 1999). The phylogenetic trees of multi-loci and single-locus were conducted using Maximum Likelihood (ML) and Bayesian Inference (BI) analyses based on RAXMLGUI 2.0 (Edler et al. 2021) and MRBAYES v. 3.1.2 (Ronquist and Huelsenbeck 2003), respectively. To determine the optimal partitioning strategy and substitution models, we used PartitionFinder 1.1.1 (Lanfear et al. 2012) with the greedy search algorithm and the Bayesian Information Criterion (BIC). Statistical supports were calculated using nonparametric bootstrapping with 1,000 replicates ML analysis (ML bootstrap: MLB). For BI analyses, using selected models and four chains were performed and stopped until the standard deviation of the split frequencies fell below 0.01 and the effective sample size (ESS) values > 200. TRACER v 1.7 (Rambaut et al. 2018) was used to monitor the chain convergence. Trees were sampled every 100 generations. Subsequently, trees were summarized and statistical supports were obtained by using the “sump” and “sumt” commands in MrBayes by discarding the first 25% generation as burn-ins. The Bayesian posterior probabilities (BPP) of internodes were estimated based on the majority rule consensus with the remaining trees.

**Table 1.** Taxa included in molecular phylogenetic analyses and their GenBank accession numbers of ITS, nrLSU, and *TEF*1*α* sequences. Accession numbers in bold indicate newly generated sequence in this study.

**Species**	**Voucher**	**Location**	**GenBank accession numbers**			**References**
			**ITS**	**nrLSU**	***TEF*1** *α*	
***Hohenbuehelia albomarginata***	**KUN-HKAS127375**	**China: Sichuan**	**PZ924535**	**PZ924543**	**PZ900481**	**This study**
***H. albomarginata***	**KUN-HKAS136466(Holotype)**	**China: Yunnan**	**PZ924537**	**PZ924544**	**PZ900480**	**This study**
***H. albomarginata***	**KUN-HKAS150407**	**China: Hebei**	**PZ924536**	**PZ924545**	**PZ900482**	**This study**
*H. algonquinensis*	RGT870601/12UWO	Canada: ON	KU355341	AF139950	KU355456	Consiglio et al. (2018)
*H. atrocoerulea*	AMB 18080	Hungary	KU355304	KU355389	KU355439	Consiglio 2017
*H. atrocoerulea*	AMB 18084	Italy	KU355301	KU355388	KU355436	—
*H. atrocoerulea*	WU 8016	Austria	KU355309	KU355390	KU355442	—
*H. angustata*	DAOM 195432a	Spain	MG383817	MG383827	—	—
*H. angustata*	CBS 856.85(Holotype)	Spain	MH861919	MG383826	—	—
*H. angustata*	DAOM 195379	Netherlands	MG383815	MH873608	—	—
*H. auriscalpium*	WU 14662	Italy	KU355317	KU355391	—	—
*H. boullardii*	JCM06005(Holotype)	Spain	MG553637	MG553644	—	Consiglio and Setti (2018)
*H. canadensis*	DAOM 158848	Canada: ON	KU355356	—	—	Consiglio et al. (2018)
*H. canadensis*	DAOM 195907	Canada: ON	KY124255	—	—	Consiglio et al. (2018)
*H. canadensis*	DAOM 46785(Holotype)	Canada: AB	KY124253	—	—	Consiglio et al. (2018)
*H. canadensis*	RGT940206/01 UWO	Canada: AB	AF139960	AF139960	—	Consiglio et al. (2018)
*H. carlothornii*	RGT990707/02 AMB 18106	Costa Rica	KY698012	KY698013	—	Consiglio et al. (2018)
*H. carlothornii*	03-RGTSN-522a UWO	Costa Rica	EF409758	EF409758	—	Consiglio et al. (2018)
*H. carlothornii*	03-RGTSN-571 UWO	Costa Rica	EF409759	EF409759	—	Consiglio et al. (2018)
*H. carlothornii*	RGT040611/01 INBio (culture UWO)	Costa Rica	EF409756	EF409756	—	Consiglio et al. (2018)
*H. cepistipes*	GXAASwsw0533	China	ON025119	OP835915	—	Qi et al. (2023)
*H. cepistipes*	GXAASwsw0534	China	ON025120	OP835916	—	Qi et al. (2023)
*H. cyphelliformis*	Z+ZT 994(Epitype)	Italy	KU355325	KU355393	—	—
*H. cyphelliformis*	AMB 18085	Italy	KU355324	KU355392	—	—
*H. faerberioides*	Mertens	Spain	MG553638	MG553645	—	—
*H. flabelliformis*	MFLU22 – 0008(Type)	Thailand	OP236779	OM521957	OM714821	—
*H. flabelliformis*	MFLU22 - 0009	Thailand	OP236780	—	—	—
*H. fluxilis*	WU 29608(Neotype)	Austria	KU355326	—	—	Consiglio 2017
*H. filicina*	LE F-332233	Russia	OQ102433	—	—	Kalinina et al. (2023)
*H. filicina*	LE F-332234(Holotype)	Russia	OQ102434	—	—	Kalinina et al. (2023)
***H. guizhouensis***	**KUN-HKAS111682(Holotype)**	**China: Guizhou**	**PZ924538**	**—**	**—**	**This study**
***H. guizhouensis***	**KUN-HKAS154712**	**China: Yunnan**	**PZ924539**	**PZ924546**	**PZ900484**	**This study**
***H. guizhouensis***	**KUN-HKAS62155**	**China: Yunnan**	**PZ924541**	**PZ924547**	**PZ900483**	**This study**
***H. guizhouensis***	**KUN-HKAS41982**	**China: Yunnan**	**PZ924540**	**PZ924548**	**—**	**This study**
*H. grisea*	01-RGTSN-523 UWO	Costa Rica	EF409761	EF409761	—	—
*H. grisea*	MCVE 27293	Italy	KU355329	KU355394	KU355447	Direct Submission
*H. grisea*	MCVE 23350	Spain	MH137807	MH137834	—	—
*H. grisea*	FH 3421	USA: IN	KU355328	—	KU355446	—
*H. ilerdensis*	Roux 3924	Spain	MG553639	MG553646	—	Consiglio and Setti (2018)
*H. incrustata*	YSU-F-05021(Holotype)	Russia: KHMAO	PP058161	—	—	Ageev et al. (2025)
*H. incrustata*	YSU-F-04996(Paratype)	Russia: KHMAO	PP058160	—	—	Ageev et al. (2025)
*H. josserandii*	P. K. EG10-812-T-F	Germany	KU355353	KU355403	—	Consiglio and Setti (2018)
*H. josserandii*	Roux 3270(Type)	France	KU355354	KU355404	—	Consiglio and Setti (2018)
*H. komarnitzkyi*	LE 17692 (Holotype)	Turkmenistan	OM219068	OM219072	—	Vlasenko et al. 2022
*H. komarnitzkyi*	LE 5606 (Isotype)	Tajikistan	OM219069	OM219073	—	Vlasenko et al. 2022
*H. lageniformis*	MFLU22 – 0010(Type)	Thailand	OP236781	OM521958	OM763737	Phonemany M.et al.2023
*H. lageniformis*	MFLU22 – 0012	Thailand	OP236783	OM521959	—	Phonemany M.et al.2023
*H. leightonii*	WU 5846	Spain	MG553640	MG553647	—	—
*H. leightonii*	WU 6004	Austria	MH137809	MH137835	—	Consiglio and Setti (2018)
*H. leightonii*	P. K. LP 311203-9	Spain	MH137810	MH137836	—	—
*H. longipes*	LIP 0400317	Italy	KU355333	KU355396	—	Direct Submission
*H. longzhouense*	GXAASwsw10126	China	ON025121	OP835913	—	Qi et al. (2023)
*H. longzhouense*	GXAASwsw10127	China	ON025122	OP835914	—	Qi et al. (2023)
*H. mastrucata*	TRTC 152314	Italy	KU355336	KU355397	—	—
*H. mustialensis*	DAOM 46374	Canada: AB	KY124252	—	—	Consiglio et al. (2018)
*H. nakhonphanomensis*	BBH 47869(Type)	—	OP458815	OP458820	OP796851	—
*H. nakhonphanomensis*	BBH 49789	—	OR598703	OR598704	—	—
*H. nimueae*	RGT 871128/01 UWO	Canada: ON	KY679144	KY679144	—	Consiglio et al. (2018)
*H. nimueae*	RGT 970530/01 UWO	USA: WY	KY679145	KY679145	—	Consiglio et al. (2018)
*H. portegna*	A27	China	KC414247	—	—	—
*H. portegna*	HE2904	China	KC505559	—	—	—
*H. portegna*	Q7	China	KC414261	—	—	—
*H. portegna*	J.E. Wright 1136 BAFC	Argentina	AF139959	AF139959	—	—
*H. petaloides*	TFB14021	USA	KP026226	KP026217	—	—
*H. reniformis*	HMJAU7091	China	GQ142024	GQ142041	—	Direct Submission
***H. striata***	**KUN-HKAS141298(Holotype)**	**China: Yunnan**	**PZ924534**	**PZ924542**	**PZ900485**	**This study**
***H. striata***	**KUN-HKAS107615**	**China: Yunnan**	**PZ924533**	**—**	**—**	**This study**
*H. thornii*	AMB 18086(Holotype)	Italy	KU355342	KU355400	—	Consiglio and Setti (2018)
*H. thornii*	WU 21790	Portugal	KU355343	MG383831	—	Consiglio and Setti (2018)
*H. thornii*	LIP 0401312	Spain	MG383823	—	—	—
*H. tomentosa*	HMJU00101	China	MK583558	MN947621	—	Xu et al. (2023)
*H. tomentosa*	HMJU00102	China	MK583564	MN382128	—	Xu et al. (2023)
*H. tremula*	WU 16294	Italy	KU355359	KU355407	—	Direct Submission
*H. tremula*	DAOM 180808	Italy	KU355357	KU355405	—	—
*H. tremula*	M 0223665(Epitype)	Italy	KU355358	KU355406	—	Consiglio 2017
*H. unguicularis*	Z+ZT 1112	Italy	KU355361	KU355408	—	Direct Submission
*H. unguicularis*	CBS 855.85(Type)	Canada: Ontario	MH861918	MH873607	—	Xu et al. 2023
*H. valesiaca*	PDG 311005	Italy	KU355339	KU355398	—	—
*H. valesiaca*	Roux 2975	France	KU355340	KU355399	—	Consiglio and Setti (2018)
*H. valesiaca*	CL 990909/04	Italy	MH137819	MH137841	—	Consiglio and Setti (2018)
*H. valesiaca*	JCS 600 B	Spain	MH137820	MH137842	—	—
*H. wilhelmii*	WU 7726	Austria	KU355300	KU355387	—	Consiglio and Setti (2018)
*H. wilhelmii*	BRNM 576483	Spain	MG383824	MG383832	—	Direct Submission
*H. wilhelmii*	Z+ZT n. 1154(Holotype)	France: Ottmarsheim	MF494947	MF494948	—	Consiglio and Setti (2018)
*Pleurotus ostreatus*	TENN 53662 (= AFTOL-ID 564)	Austria	AY854077	AY645052	AY883432	Li et al. (2017)
*Tuoliensis*	CCMSSC03105	China	KU612906	—	KU612970	Li et al. (2017)
*P. citrinopileatus*	KUN-HKAS85965	China	KP867920	KP867911	KP867899	Li et al. (2017)
*P. djamor*	KUN-HKAS94069	Sri Lanka	KX904522	—	KX840308	Li et al. (2017)

### Morphological studies

Field notes and digital photos are used to describe macroscopic features. The color codes are based on work by Kornerup and Wanscher (1981). Microscopic observations were on dried specimens mounted on 5% KOH and stained with Congo red when necessary. “[n/m/p]” denotes the measurement of n basidiospores from m basidiomata of p collections. The size of basidiospores is represented as “(a–) b–c (–d)”. At least 90% of the measured values are present in the range b–c. Parentheses are used to indicate extreme values in a and d. *x̄* means average length of basidiospores ± sample standard deviation × average width of basidiospores ± sample standard deviation. Q is a basidiospore’s side view length to width ratio. Qm indicates average Q of all basidiospores measured ± sample standard deviation. SigmaPlot 10.0 was used to determine these numbers (Systat Software, San Jose, CA). With a phase contrast objective (× 1000), line drawings of the new species were illustrated by hand under a microscope.

## Results

### Phylogenetic analyses

**Figure 1.**
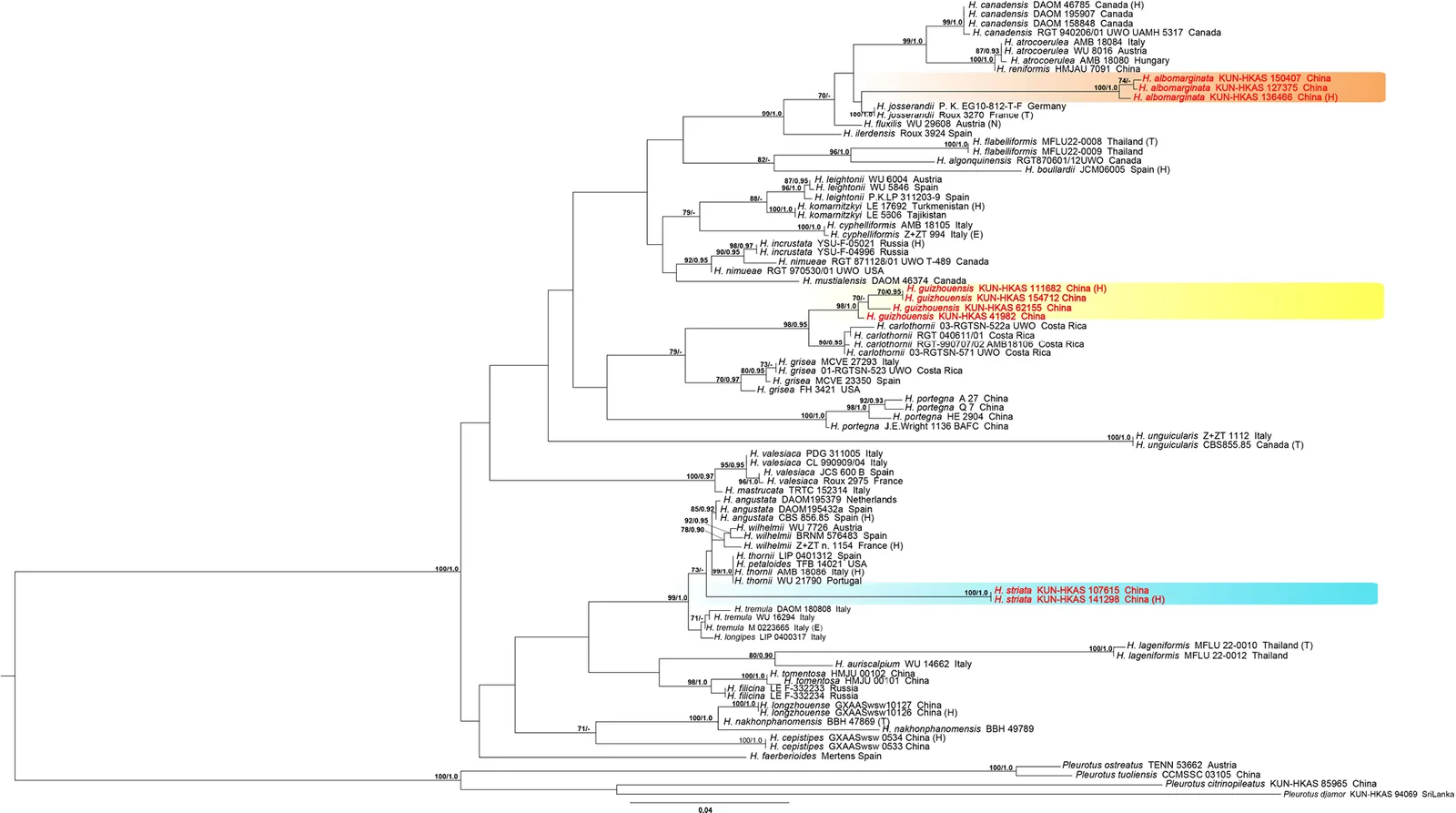
The maximum likelihood (ML) tree of *Hohenbuehelia* inferred from the multi-locus dataset (ITS, nrLSU and *TEF*1*α*). ML bootstrap values over 70% (MLB ≥ 70%) and Bayesian posterior probabilities over 0.90 (BPP ≥ 0.90) are shown above or beneath individual branches. Sequences from type, holotype or epitype specimens are marked with (T), (H) or (E) respectively, the three new species are indicated in red bold face.

In total, 22 sequences generated in this study were included in the single-locus and multi-loci datasets. In the three single-locus datasets, GTR+GAMMA model was the most appropriate substitution model for ITS, GTR+GAMMA+I model was the most appropriate substitution model for nrLSU and *TEF*1α. The aligned three-gene matrix contained a total of 87 samples and 2242 aligned bases for each sample. Phylogenetic trees of the species complex were constructed on the basis of these single-locus and three-gene matrices. Considering that the topology of phylogenetic trees based on three-gene dataset generated from ML and BI analyses were almost identical, while statistical supports showed slight differences, only the tree inferred from ML analysis was displayed (Fig. 1).

**Figure 2.**
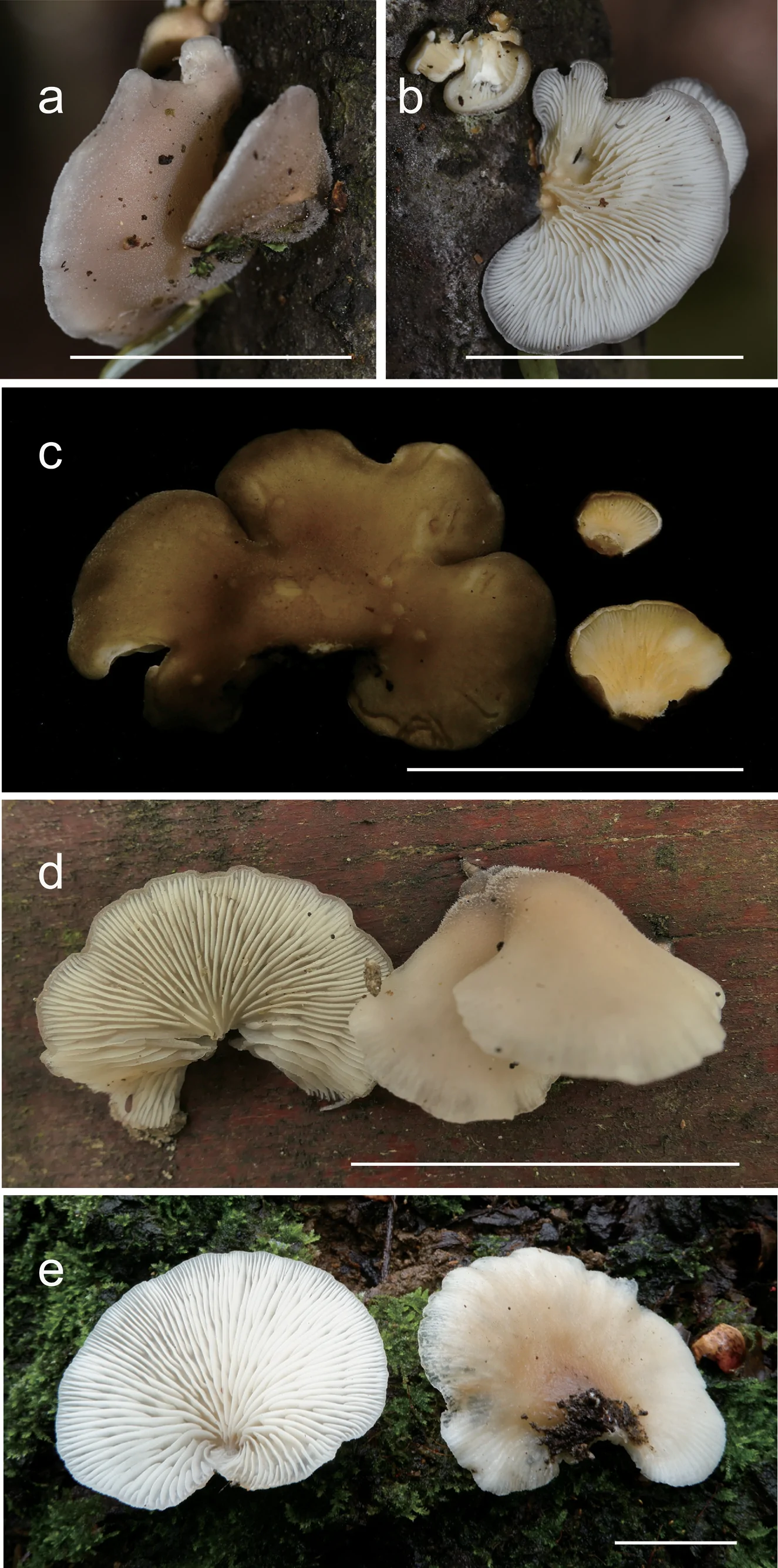
Fresh basidiomata of the three new *Hohenbuehelia* species. **a–c**. *H. albomarginata* (**a, b** KUN-HKAS127375, **c** Holotype, KUN-HKAS136466); **d**. *H. guizhouensis* (Holotype, KUN-HKAS111682); **e**. *H. striata* (Holotype, KUN-HKAS141298). Scale bars: 2 cm.

In the concatenated multi-locus phylogeny, each of the three new species forms a strongly supported monophyletic clade (MLB/BPP = 100%/1.0 for each). The sister-clade relationships between these species and their closest allies receive variable support (ranging from MLB 73% to 100%), indicating that while the species themselves are robustly delimited, the deeper internodal relationships within *Hohenbuehelia* may require additional molecular markers and broader taxon sampling for full resolution. *Hohenbuehelia albomarginata* is related to *Hohenbuehelia fluxilis* (Fr.) P.D. Orton, *Hohenbuehelia josserandii* Consiglio & Setti, *Hohenbuehelia reniformis* (G. Mey.) Singer, *Hohenbuehelia atrocoerulea* (Fr.) Singer and *Hohenbuehelia canadensis* Consiglio, Setti & Thorn. *Hohenbuehelia striata* clusters together with *Hohenbuehelia thornii* Consiglio & Setti, *Hohenbuehelia petaloides* (Bull.) Schulzer, *Hohenbuehelia wilhelmii* Consiglio & Setti and *Hohenbuehelia angustata* (Berk.) Singer. *Hohenbuehelia guizhouensis* forms a monophyletic clade as a sister lineage to *Hohenbuehelia carlothornii* Consiglio, Setti & Thorn. (Fig. 2).

## Taxonomy

### Hohenbuehelia albomarginata

Taxon classificationFungiAgaricalesPleurotaceae

J.Li & Y.Y.Cui sp. nov.

BC670C5A-AEE6-587C-ACDC-2DA6EDF6ED9B

Fungal Names: FN 574009

#### Etymology.

albo- = albus, -marginata = marginis, referring to its pileus often with white margin.

#### Diagnosis.

It differs from *H. paraguayensis* by having a gray-brown pileus with white margin and white tomentum.

#### Type.

China • Yunnan Province: Baoshan, Tengchong, on wood in subtropical broad-leaved forests, altitude 1700 m, 23 July 2022, GLG-FXP153 (**holotype**, KUN-HKAS136466).

#### Description.

***Basidiomata*** gregarious to imbricate, pleurotoid, laterally attached by a pseudostipe. ***Pileus*** 0.5–2.5 cm wide, fan-shaped to dimidiate, moist, gray-brown (3D2–5) to brown (3D3–6), often white (1A1) at margin, frosted with white (1A1) tomentum, becoming farinose towards margin. ***Context*** thin. ***Lamellae*** decurrent, crowded, white (1A1) when young, becoming yellowish (1A2) to yellow (3A3) from base to margin with age. ***Pseudostipe*** very short and stout, laterally attached, flattened, widest at the apex, concolorous with lamellae.

**Figure 3.**
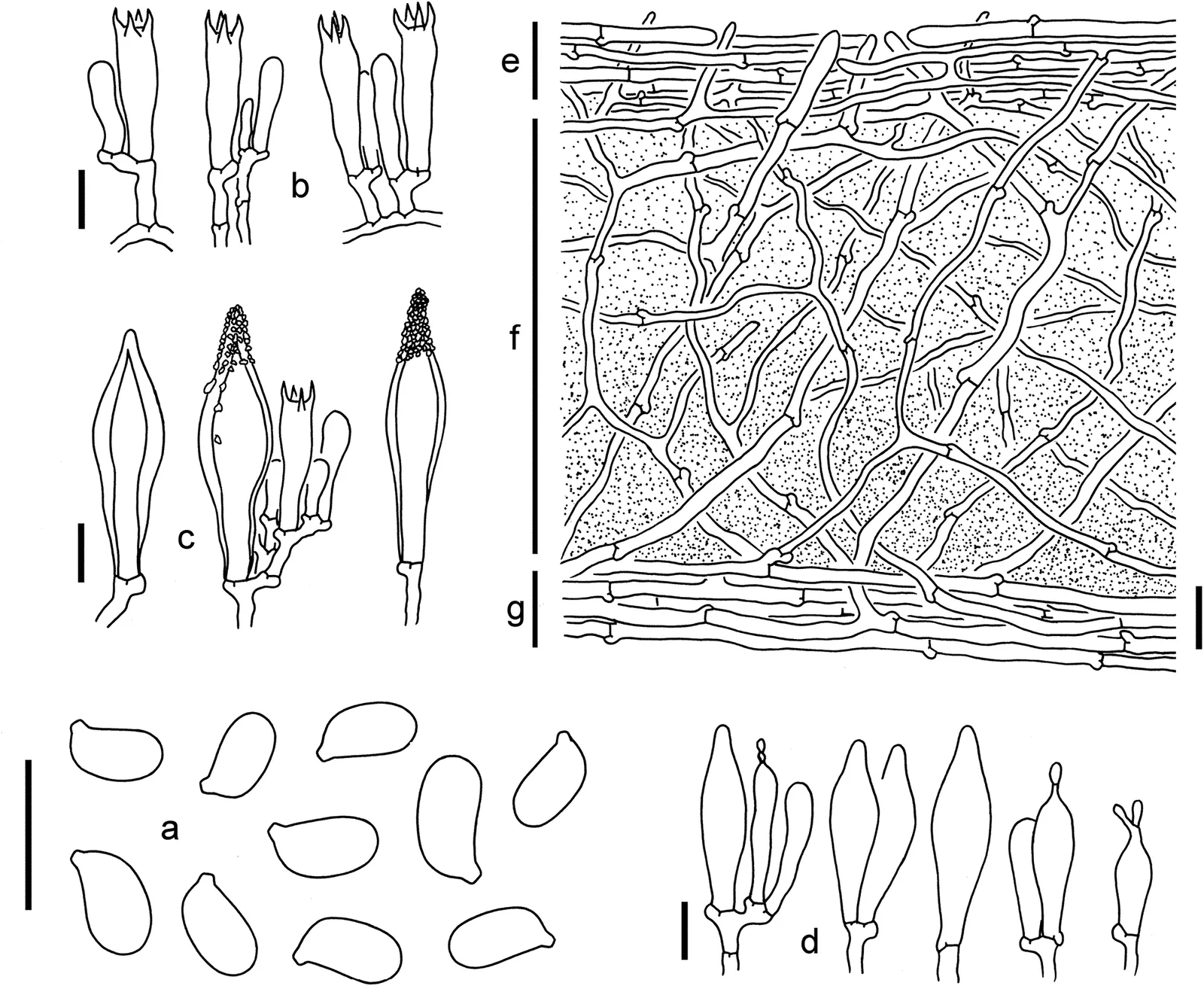
Microscopic features of *Hohenbuehelia albomarginata* (KUN-HKAS136466). **a**. Basidiospores; **b**. Hymenium and subhymenium; **c**. Pleurocystidia; **d**. Cheilocystidia; **e**. Pileipellis; **f**. Gelatinous zone; **g**. Pileitrama. Scale bars: 10 µm (**a–d**); 20 µm (**e–g**).

***Basidiospores*** [33/1/1] (5.5–) 6–7.5 (–8) Í 3–4.5 µm (mean length = 6.6 µm, mean width = 3.7 µm), Q = (1.45–) 1.62–2 (–2.2), Qm = 1.81 ± 0.17, mostly elongate, sometimes ellipsoid or cylindrical, non-amyloid, thin-walled, smooth. ***Basidia*** 17–37 Í 4.5–6.5 µm, clavate to subclavate, 4-spored; sterigmata 3–5 µm long; basal septa with clamps. ***Pleurocystidia*** metuloid, subfusoid to fusoid, with a narrow base, 45–55 Í 10–14 µm, thick-walled, sometimes with a lanceolate apical part and partly covered with refringent crystalline granules. ***Cheilocystidia*** clustered in groups along the lamellar edge, mostly fusoid to fusoid-ventricose, sometimes clavate with one or two swellings protruding from the apical portion, with a neck and capitulum, thin-walled. ***Pileipellis*** a cutis, composed of radially arranged, 2–6 µm wide, thin-walled, colorless to yellowish, filamentous hyphae. ***Gelatinous zone*** composed of interwoven to more or less erect arranged hyphae, 2–4 µm wide. ***Pileitrama*** a compact cutis, composed of radially arranged, 2–7 µm wide, thin-walled, filamentous to cylindrical hyphae. ***Clamps*** ubiquitous (Fig. 3).

#### Ecology.

Single or scattered, on wood in subtropical broad-leaved forests with trees of *Fagaceae* and warm-temperate deciduous broad-leaved forests.

#### Distribution.

Basidioma occurring in summer to autumn. Known from Southwest and North China.

#### Additional specimens examined.

China • Sichuan Province: Aba Tibetan and Qiang Autonomous Prefecture, Jiuzhaigou, on wood in subtropical broad-leaved forests, altitude 2390 m, 19 July 2020, JLK1084 (KUN-HKAS127375). • Hebei Province: Pingshan County, Tuoliang Mountain National Forest Park, on wood in warm-temperate deciduous broad-leaved forests, altitude 1620 m, 11 September 2024, LZW638 (KUN-HKAS150407).

#### Notes.

*Hohenbuehelia albomarginata* has small, gray-brown to brown basidiomata, a pileus frosted with white tomentum, becoming farinose towards margin, and elongate basidiospores. Our phylogenetic analysis places it within a clade including *H. fluxilis*, *H. josserandii*, *H. canadensis*, *H. reniformis* and *H. atrocoerulea*. Indeed, *H. josserandii*, *H. reniformis* and *H. atrocoerulea* all have small, gray-brown to brown basidiomata with tomentose pilei (Li et al. 2014; Consiglio et al. 2018; Holec and Zehnálek 2020). However, *H. josserandii* can be distinguished from *H. albomarginata* by its darker pileus, sparser lamellae, slightly longer basidiospores (7.5–9 × 3.5–4 μm), and its occurrence on dead wood of *Abies* (Holec and Zehnálek 2020). *Hohenbuehelia reniformis* has cylindrical basidiospores (Li et al. 2014). *Hohenbuehelia atrocoerulea* is characterized by a pileus covered with dense, pale yellowish-brown to dark blue tomentum (Li et al. 2014). In China, *Hohenbuehelia atrocoerulea* is presently only known from subtropical areas of Hainan Province (Li et al. 2014). *Hohenbuehelia canadensis* is further distinguished by its black pileus with gray tomentum, cream to pale grayish-yellow lamellae, and its occurrence on conifer wood in North America (Consiglio et al. 2018). *Hohenbuehelia fluxilis* is the only species in the lineage with a translucent-striate pileus margin and an off-white to pale gray-brown pileus (Li et al. 2014).

Morphologically, *H. unguicularis* (Fr.) O.K. Mill, *H. nimueae* Consiglio, Setti & Thorn, *H. tomentosa* J.Z. Xu, *H. paraguayensis* (Speg.) Singer, *H. portegna* (Speg.) Singer and *H. incrustata* Ageev, Bulyonk., Zvyagina & Filippova may be confused with *H. albomarginata* due to their gray-brown pileus frosted with tomentum and ellipsoid basidiospores (Li et al. 2014; Silva-Filho and Cortez 2017; Consiglio et al. 2018; Xu et al. 2023; Ageev et al. 2025). However, *H. unguicularis* has a smaller, horseshoe-shaped pileus, grayish-white lamellae, and is commonly found on wood of *Pinus massoniana* (Li et al. 2014). *Hohenbuehelia nimueae* has lamellae radiating from the point of attachment, ellipsoid to phaseoliform basidiospores, and its occurrence singly on Salix branches in Sphagnum bogs (Consiglio et al. 2018). *Hohenbuehelia tomentosa* differs from *H. albomarginata* in having a darker pileus, a longer subcentral stipe (2–4 × 0.7–0.9 cm) with longitudinally fibrous striations, and its abundance in temperate regions of northeastern China (Xu et al. 2023). *Hohenbuehelia paraguayensis* and *H. portegna* differs significantly from *H. albomarginata* in having a hirsute to pubescent, slightly lobed and striate pileus; moreover, *H. portegna* has longer basidiospores (7–11.5 × 3–5 μm) (Silva-Filho and Cortez 2017).

### Hohenbuehelia guizhouensis

Taxon classificationFungiAgaricalesPleurotaceae

J.Li & Y.Y.Cui sp. nov.

8A15505B-7046-5220-8C9E-5026FB1AA7B2

Fungal Names: FN 574010

#### Etymology.

guizhouensis = of Guizhou, referring to Guizhou Province, China, where the type specimen was collected.

#### Diagnosis.

Related to *H. portegna*, but differs by lamellae radiating from the point of attachment, and elongate, ellipsoid to subreniform basidiospores.

#### Type.

China • Guizhou Province: Qiandongnan, Leishan, on the fallen wood, altitude 1820 m, 26 July 2018, WXH4919 (**holotype**, KUN-HKAS111682).

#### Description.

***Basidiomata*** scattered to imbricate, pleurotoid, laterally attached without pseudostipe. ***Pileus*** 0.5–1.8 cm wide, initially reniform, later fan-shaped to dimidiate, moist, gray-brown (5B3–4) to brown (6C4–5), often white (1A1) at margin, but sometimes remaining brown (6C4–5), frosted with white (1A1) tomentum, more conspicuous near the point of attachment. ***Context*** thin. ***Lamellae*** radiating from point of attachment, moderately close, white (1A1) at first, becoming pale yellow (1B3) with age.

**Figure 4.**
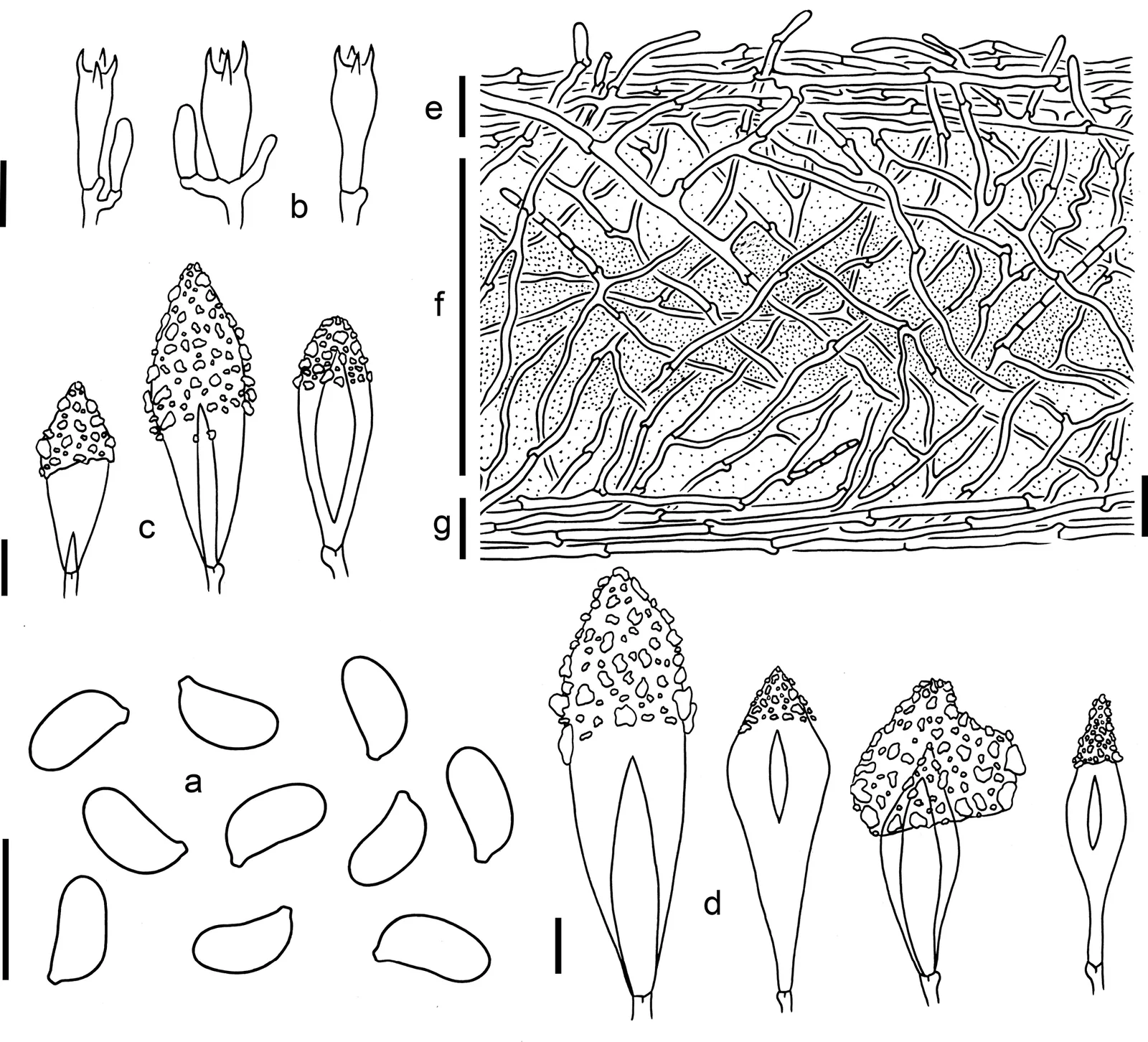
Microscopic features of *Hohenbuehelia guizhouensis* (KUN-HKAS111682, KUN-HKAS62155). **a**. Basidiospores; **b**. Hymenium and subhymenium; **c**. Pleurocystidia; **d**. Cheilocystidia; **e**. Pileipellis; **f**. Gelatinous zone; **g**. Pileitrama. Scale bars: 10 µm (**a–d**); 20 µm (**e–g**).

***Basidiospores*** [48/2/2] 6–8 Í 3–4 (–4.5) µm (mean length = 7.1 µm, mean width = 3.6 µm), Q = (1.64–) 1.78–2.26 (–2.34), Qm = 2 ± 0.14, mostly elongate, sometimes ellipsoid or subreniform, non-amyloid, thin-walled, smooth. ***Basidia*** 13–25 Í 5–7.5 µm, subclavate to clavate, 4-spored; sterigmata 3–5 µm long; basal septa with clamps. ***Pleurocystidia*** metuloid, subfusoid to fusoid, sometimes with a narrow base, 47–80 Í 10–22 µm, thick-walled, with a lanceolate apical part and partly covered with refringent crystalline granules. ***Cheilocystidia*** metuloid, fusoid, rarely broadly fusoid, 33–52 Í 11–15 µm, thick-walled, with a lanceolate apical part and partly covered with refringent crystalline granules. ***Pileipellis*** a cutis, composed of radially arranged, 2–5 µm wide, thin-walled, colorless, filamentous to cylindrical hyphae. ***Gelatinous zone*** composed of interwoven to more or less erect arranged hyphae, 1–4 µm wide. ***Pileitrama*** a cutis, composed of radially, 2–5 µm wide, thin-walled, filamentous to cylindrical hyphae. ***Clamps*** ubiquitous (Fig. 4).

#### Ecology.

Single or scattered, on wood in subtropical broad-leaved forests with trees of *Fagaceae*.

#### Distribution.

Basidioma occurring in summer to autumn. Known from Southwest China.

#### Additional specimens examined.

China • Yunnan Province: Dali Bai Autonomous Prefecture, Binchuan County, on the dead branches, altitude 3240 m, 16 August 2010, Li shuhong-BC-17 (KUN-HKAS62155). • Yunnan Province: Baoshan City, Tengchong County, on wood in subtropical broad-leaved forests, altitude 1850 m, 6 October 2002, WHC-211 (KUN-HKAS41982). • Yunnan Province: Chuxiong Yi Autonomous Prefecture, Lufeng County, on the dead branches, altitude 2300 m, 24 July 2023, GXL0095 (KUN-HKAS154712).

#### Notes.

*Hohenbuehelia guizhouensis* has small, gray-brown to brown basidioma, a pileus frosted with white tomentum, and mostly elongate, sometimes ellipsoid or subreniform basidiospores. *Hohenbuehelia guizhouensis* and *H. carlothornii* are phylogenetically closely related, forming a well-supported clade (bootstrap = 98%). However, *H. guizhouensis* can be distinguished from *H. carlothornii* by its elongate, ellipsoid or subreniform basidiospores. Ecologically, *H. guizhouensis* is primarily found in subtropical broad-leaved forests, whereas *H. carlothornii* is isolated from soil and litter, and fruits on dead angiosperm wood in tropical wet forest and premontane rain forest in Costa Rica (Consiglio et al. 2018).

Morphologically, *H. guizhouensis* resembles *H. nimueae*, *H. portegna*, and *H. incrustata*, particularly in the field, often leading to confusion. However, microscopic examination shows that *H. guizhouensis* has lamellae radiating from the point of attachment, elongate, ellipsoid or subreniform basidiospores (6–8 × 3–4 µm), and subclavate to clavate basidia (13–25 × 5–7.5 µm), which distinguished it from *H. portegna* and *H. incrustata* (Silva-Filho and Cortez 2017; Ageev et al. 2025). *Hohenbuehelia nimueae* differs in having cupulate-pendent to dimidiate basidiomata and occurring singly on *Salix* branches in Sphagnum bogs (Consiglio et al. 2018).

### Hohenbuehelia striata

Taxon classificationFungiAgaricalesPleurotaceae

J.Li & Y.Y.Cui sp. nov.

16C8700F-470A-5826-8800-967C677CE143

Fungal Names: FN 574011

#### Etymology.

striata = striate, referring to its translucent-striate pileal margin.

#### Diagnosis.

Similar to *H. aurantiocystis*, but differs from the latter in having ellipsoid basidiospores.

#### Type.

China • Yunnan Province: Baoshan, Longling County, on wood in subtropical broad-leaved forests, altitude 1470–1530 m, 27 July 2023, GLG-FXP1528 (**holotype**, KUN-HKAS141298).

#### Description.

***Basidiomata*** gregarious, laterally or dorsally attached without stipe or pseudostipe. ***Pileus*** 4.5–6 cm wide, fan-shaped, dimidiate to orbicular, very faintly translucent-striate near margin, glabrous, very finely frosted or nearly glabrous at margin, moist, shiny, brown (3E4–7), yellow-brown (4D4–7) to brownish (2C2–5), becoming dirty white (2B2) to white (1A1) towards margin. ***Context*** thin. ***Lamellae*** radiating from point of attachment, moderately close, white (1A1) to dirty white (2B1–3).

**Figure 5.**
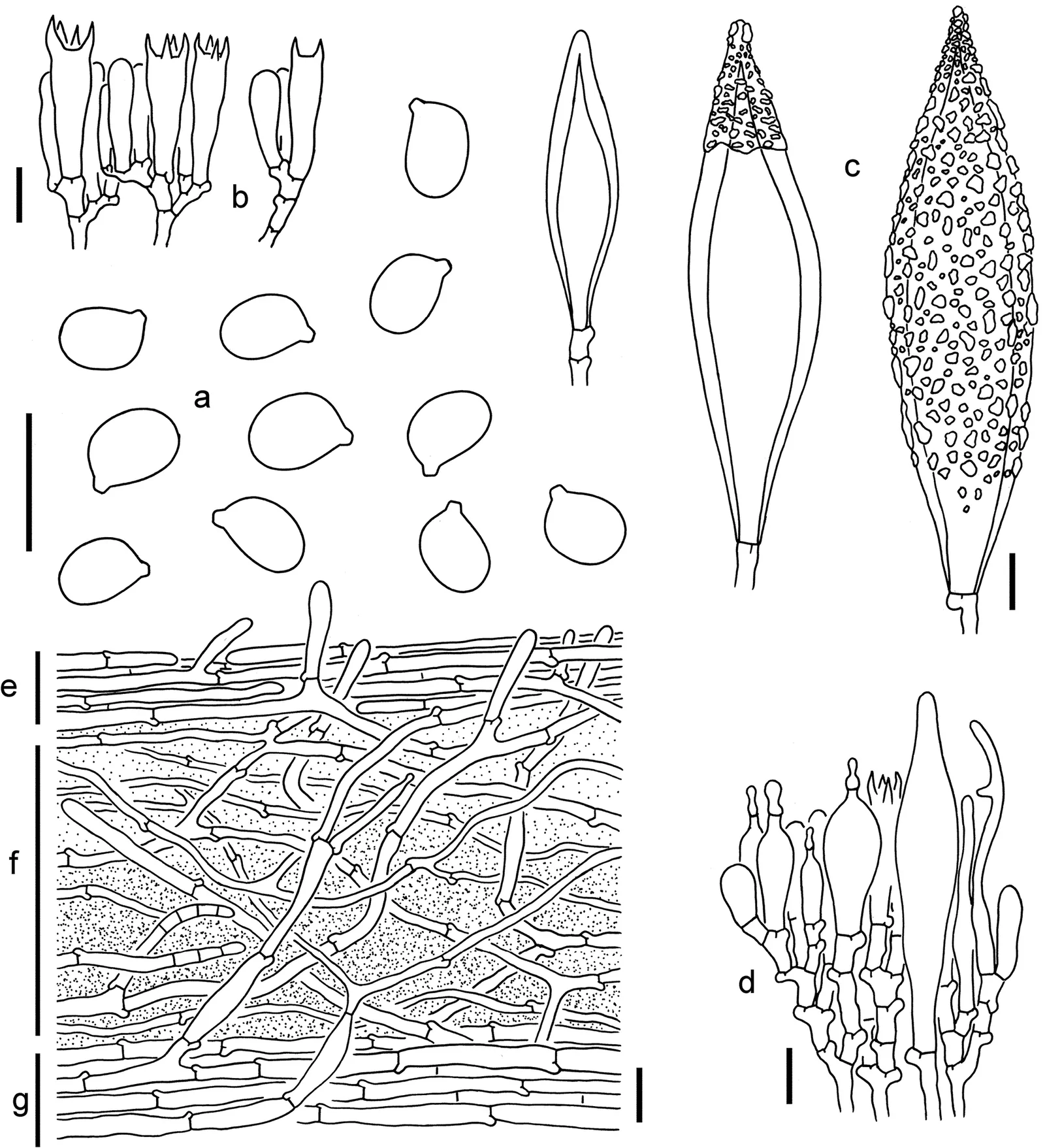
Microscopic features of *Hohenbuehelia striata* (KUN-HKAS141298). **a**. Basidiospores; **b**. Hymenium and subhymenium; **c**. Pleurocystidia; **d**. Cheilocystidia; **e**. Pileipellis; **f**. Gelatinous zone; **g**. Pileitrama. Scale bars: 10 µm (**a–d**); 20 µm (**e–g**).

***Basidiospores*** [20/1/1] 5.5–6.8 (–7.5) Í 4–5.5 µm (mean length = 6.3 µm, mean width = 4.6 µm), Q = 1.25–1.48 (–1.55), Qm = 1.36 ± 0.08, mostly ellipsoid, sometimes broadly ellipsoid, non-amyloid, thin-walled, smooth. ***Basidia*** 20–30 Í 6–7.5 µm, clavate, mostly 4-spored, sometimes 2-spored; sterigmata 5–7 µm long; basal septa with clamps. ***Pleurocystidia*** metuloid, subfusoid, with a narrow base, 45–110 Í 15–25 µm, thick-walled (3–6 µm), mostly with a lanceolate apical part and partly covered with refringent crystalline granules, sometimes glabrous and without crystalline granules. ***Cheilocystidia*** clustered in groups along the lamellar edge, mostly clavate-capitate with one or two swellings protruding from the apical portion, with a neck and capitulum, sometimes fusoid-ventricose with a long neck, thin-walled. ***Pileipellis*** a cutis, composed of radially arranged to interwoven, 2–6 µm wide, thin-walled, filamentous hyphae. ***Gelatinous zone*** composed of interwoven to more or less erect arranged hyphae, 2–7 µm wide. ***Pileitrama*** a compact cutis, composed of radially arranged, 3–7 µm wide, thin-walled, filamentous to cylindrical hyphae. *Clamps* ubiquitous (Fig. 5).

#### Ecology.

Single or scattered, on wood in subtropical broad-leaved forests with trees of *Fagaceae*.

#### Distribution.

Basidioma occurring in summer. Known from Southwest China.

#### Additional specimen examined.

China • Yunnan Province: Kunming City, Panlong County, Kunming Institute of Botany, Chinese Academy of Science, on wood in subtropical broad-leaved forests, altitude 1960 m, 22 July 2020, Yang6330 (KUN-HKAS107615).

#### Notes.

*Hohenbuehelia striata* is characterized by brown, glabrous basidiomata, laterally or dorsally attached, without a stipe or pseudostipe, and ellipsoid to broadly ellipsoid, smooth basidiospores. Phylogenetically, it clusters with *H. angustata*, *H. wilhelmii*, *H. thornii* and *H. petaloides* in our phylogenetic analysis. However, *H. striata* can be distinguished from *H. angustata* by its brown basidiomata and larger ellipsoid basidiospores (Li et al. 2014). *Hohenbuehelia thornii* has a small, off-white pileus and cylindrical basidiospores (Q = 2.08–2.42) (Consiglio 2017; Consiglio and Setti 2018). *Hohenbuehelia petaloides* is characterized by a yellow, non-striate, spathulate pileus and decurrent lamellae (Schulzer von Müggenburg et al. 1866). Molecular data support a well-defined clade of European isolates, which was described by Consiglio and Setti (2018) as *H. wilhelmii*.

On the other hand, *H. striata* is morphologically similar to *H. grisea* (Peck) Singer, *H. bonii* A.M. Ainsw, *H. auriscalpium* (Maire) Singer, *H. aurantiocystis* Pegler and *H. reniformis*, all of which are characterized by a brown pileus. However, *H. grisea*, *H. bonii*, *H. auriscalpium* and *H. reniformis* differ in having a white tomentose pileus and cylindrical to ellipsoid basidiospores (Liu et al. 2010; Li et al. 2014; Ainsworth et al. 2017; Holec and Zehnálek 2020). *Hohenbuehelia aurantiocystis* differs from *H. striata* in having a non-striate pileus margin, a pale orange context, and broad to broadly ellipsoid basidiospores (Li et al. 2014).

## Discussion

Only a few morphological characters of *Hohenbuehelia* species were available for taxonomy, and the color of the fruiting body varied in a broad range (Thorn and Barron 1986; He 1992; Courtecuisse et al. 1999; Liu and Bau 2009). This underscores the necessity of integrating molecular methods for accurate classification and species identification. Recent advancements in molecular analyses, particularly multi-locus phylogenetic approaches, have significantly accelerated the discovery of new *Hohenbuehelia* species (Consiglio et al. 2018; Vlasenko et al. 2022; Kalinina et al. 2023; Phonemany et al. 2023; Qi et al. 2023; Xu et al. 2023; Ageev et al. 2025; Somrithipol et al. 2025). In this study, the discovery of *H. albomarginata*, *H. guizhouensis* and *H. striata* not only expands the known diversity of this genus in China but also underscores the need for broader taxon sampling and comprehensive phylogenetic analyses to fully elucidate its species richness and ecological significance. Future research should focus on exploring understudied regions and habitats to uncover additional cryptic species and refine the evolutionary relationships within this ecologically important genus.

With the three new species, the total number of the species of *Hohenbuehelia* known from China reaches 17 (He 1992; Liu and Bau 2009; Liu et al. 2010; Li et al. 2014; Qi et al. 2023; Xu et al. 2023; this study). *Hohenbuehelia petaloides* and *H. reniformis* (G. Mey.) Singer appears to be the most common taxa both by their geographical distribution, with records from nearly every region of the country, and by the total number of finds recorded under these names. In China, *Hohenbuehelia* species have been found in many provinces, such as Jilin, Neimenggu, Shanxi, Hunan, Guangdong, Hainan. Southwestern China has been widely acknowledged as a global biodiversity hotspot, renowned for its high levels of microbial diversity. Our discovery of the three species of *Hohenbuehelia* from Yunnan Sichuan and Guizhou Province adds to the growing list of fungi described from this region and highlights the importance of continued mycological surveys in under-explored habitats.

### Key to the *Hohenbuehelia* species known from China

**Table:** 

1	Basidiomata with a distinct stipe (stipe present)	**2**
–	Basidiomata sessile or with a pseudostipe (stipe absent)	**5**
2	Stipe 3–5 mm long; basidiospores globose to subglobose; context orange yellow	***H. aurantiocystis***
–	Stipe 10–40 mm long; basidiospores ellipsoid or subglobose; context not orange yellow	**3**
3	Pileus surface with gray white, brownish or gray brown tomentose; basidiospores ellipsoid, with width ca. 6.5–8 µm	***H. grisea***
–	Pileus surface with white tomentose; basidiospores subglobose, ellipsoid to oblong-ellipsoid, with width less than 5 µm	**4**
4	Pileus gray brown to nearly black, covered with dense pure white tomentum; basidiospores 5.0–7.1 × 3.6–4.4 µm, Q = 1.52–1.92, ellipsoid to oblong-ellispoid; cheilocystidia incrusted with crystals	***H. tomentosa***
–	Pileus gray to pale gray, covered with white cottony-floccose tomentum; basidiospores 4.5–6.5 × 3.0–3.5 μm, Q = 1.2–2.1, subglobose to ellipsoid; cheilocystidia without crystals	***H. cepistipes***
5	Basidiomata small, usually less than 20 mm wide	**6**
–	Basidiomata medium to large, usually more than 20 mm wide	**10**
6	Hymenium without cystidia	***H. silvana***
–	Hymenium with cystidia	**7**
7	Pileus black or nearly black	***H. nigra***
–	Pileus not black, mostly gray-brown to brown	**8**
8	Pileus without a white margin; lamellae gray white, not with yellow color in development	***H. unguicularis***
–	Pileus often white at margin; lamellae white at first, becoming yellowish to yellow with age	**9**
9	Pseudostipe distinct; cheilocystidia not metuloid	***H. albomarginata***
–	Pseudostipe absent; cheilocystidia not metuloid	***H. guizhouensis***
10	Pileus surface glabrous or nearly glabrous, sometimes with finely tomentose	**11**
–	Pileus surface with obvious tomentose	**13**
11	Pileus without transverse wrinkles and without translucent striae	***H. petaloides***
–	Pileus with transverse wrinkles or with translucent striae	**12**
12	Pileus with transverse wrinkles	***H. ingentimetuloidea***
–	Pileus without transverse wrinkles but with very faintly translucent striate near margin	***H. striata***
13	Basidiospores globose to subglobose	**14**
–	Basidiospores ellipsoid, elongate to cylindrical	**15**
14	Basidioma without a stipe; basidiospores relatively larger, 5.0–7.5 × 5.0–7.0 μm	***H. longzhousis***
–	Basidioma with a pseudostipe; basidiospores relatively smaller, 3.5–4.5 × 4–5 μm	***H. angustata***
15	Pileus surface with white or incanus velutinous, hygrophanous	***H. reniformis***
–	Pileus surface with off-white velutinous	**16**
16	Pileus nearly plane; tomentum mouse-gray to olive-green	*H. olivacea*
–	Pileus margin incurved; tomentum gray-brown, gray-purple, or pale bluish yellow brown	***H. atrocoerulea***

## Supplementary Material

XML Treatment for Hohenbuehelia albomarginata

XML Treatment for Hohenbuehelia guizhouensis

XML Treatment for Hohenbuehelia striata

## Additional information

### Artificial Intelligence (AI) use

The authors accept full responsibility for the content of the manuscript, including the disclosure of any use of AI.

No AI tools were used in the preparation of this manuscript.

### Author ORCIDs

Y.-Y. Cui https://orcid.org/0000-0003-3990-8342

### Data availability

All of the data that support the findings of this study are available in the main text.

## References

[B1] Ageev DV, Bulyonkova TM, Zvyagina EA, Filippova NV (2025) *Hohenbuehelia incrustata* sp. nov., a new species of *Hohenbuehelia* (*Pleurotaceae*, *Agaricales*) from Northwestern Siberia, Russia. Phytotaxa 683: 133–144. 10.11646/phytotaxa.683.2.4

[B2] Ainsworth AM, Suz LM, Dentinger BTM (2017) *Hohenbuehelia bonii* sp. nov. and *H. culmicola*: two pearls within the Marram Oyster. Mycology 17: 78–86. 10.1016/j.fldmyc.2016.07.004

[B3] Albertó E, Fazio A, Wright JE (1998) Reevaluation of *Hohenbuehelia nigra* and species with close affinities. Mycologia 90: 142–150. 10.1080/00275514.1998.12026891

[B4] Barron GL (1977) The nematode-destroying fungi. Canadian Biological Publications, Guelph.

[B5] Barron GL, Thorn RG (1987) Destruction of nematodes by species of *Pleurotus*. Canadian Journal of Botany 64: 774–778. 10.1139/b87-103

[B6] Consiglio G (2017) Nomenclatural novelties. Index Fungorum 326, 1. https://www.speciesfungorum.org/Names/SynSpecies.asp?RecordID=552787

[B7] Consiglio G, Setti L (2018) The genera *Hohenbuehelia* and *Resupinatus* in Europe / I generi *Hohenbuehelia* e *Resupinatus* in Europe. Monografie di Pagine di Micologia. Associazione Micologica Bresadola, Trento.

[B8] Consiglio G, Setti L, Thorn RG (2018) New species of *Hohenbuehelia*, with comments on the *Hohenbuehelia atrocoerulea*—*Nematoctonus robustus* species complex. Persoonia 41: 202–212. 10.3767/persoonia.2018.41.10 PMC6344808 30728605

[B9] Corner EJH (1994) On the Agaric genera *Hohenbuehelia* and *Oudemansiella* Part I: *Hohenbuehelia*. Gardens’ Bulletin Singapore 46: 1–47.

[B10] Courtecuisse R, Vila J, Llimona X (1999) *Hohenbuehelia ilerdensis*, a new graminicolous species from semisteppe areas of Lleida (Western Catalonia, Spain). Mycotaxon LXX: 1–5. 10.5962/p.415025

[B11] Doyle J, Doyle L (1987) A rapid DNA isolation procedure for small quantities of fresh leaf tissue. Phytochemistry Bulletin 19: 11–15.

[B12] Drechsler C (1941) Some hyphomycetes parasitic on free-living terricokms nematodes. Phytopathology 31: 773–802.

[B13] Edler D, Klein J, Antonelli A, Silvestro D (2021) raxmlGUI 2.0: A graphical interface and toolkit for phylogenetic analyses using RAxML. Methods in Ecology and Evolution 12: 373–377. 10.1111/2041-210X.13512

[B14] Hall TA (1999) BioEdit: a user-friendly biological sequence alignment editor and analysis program for Windows 95/98/NT. Nucleic Acids Symposium Series 41: 95–98.

[B15] He MQ, Zhao RL, Hyde KD, Begerow D, Kemler M, Yurkov A, McKenzie EHC, Raspé O, Kakishima M, Sánchez-Ramríez S, Vellinga EC, Halling R, Papp V, Zmitrovich VI, Buyck B, Ertz D, Wijayawardene NN, Cui BK, Schoutteten N, Liu XZ, Li TH, Yao YJ, Zhu XY, Lliu AQ, Li GJ, Zhang MZ, Ling ZL, Cao B, Antonín V, Boekhout T, Silva BDB, Crop ED, Decock C, Dima B, Dutta AK, Fell JW, Geml J, Ghobad-Nejhad M, Giachini AJ, Gibertoni TB, Gorjón SP, Haelewaters D, He SH, Hodkinson BP, Horak E, Hoshino T, Justo A, Lim YW, Menolli N, Mešić A, Moncalvo JM, Mueller GM, Nagy LG, Nilsson RH, Noordeloos M, Nuytinck J, Orihara T, Ratchadawan C, Rajchenberg M, Silva-Filho AGS, Sulzbacher MA, Tkalčec Z, Valenzuela R, Verbeken A, Vizzini A, Wartchow F, Wei TZ, Weiß M, Zhao CL, Kirk PM (2019) Notes, outline and divergence times of *Basidiomycota*. Fungal Diversity 99: 105–367. 10.1007/s13225-019-00435-4

[B16] He X (1992) A new species and a new record of *Tricholomataceae* from China. Acta Mycologica Sinica 11: 18–22. 10.13346/j.mycosystema.1992.01.004

[B17] Holec J, Zehnálek P (2020) Taxonomy of *Hohenbuehelia auriscalpium*, *H. abietina*, *H. josserandii*, and one record of *H. tremula*. Czech Mycology 72: 199–220. 10.33585/cmy.72204

[B18] Kalinina LB, Ageev DV, Bulyonkova TM (2023) *Hohenbuehelia filicina* sp. nov. (*Agaricales*, *Basidiomycota*), from Southwestern Siberia, Russia. Phytotaxa 600: 272–280. 10.11646/phytotaxa.600.5.2

[B19] Katoh K, Standley DM (2013) MAFFT multiple sequence alignment software version 7: improvements in performance and usability. Molecular Biology and Evolution 30: 772–780. 10.1093/molbev/mst010 PMC3603318 23329690

[B20] Kirk PM, Cannon PF, David JC, Stalpers JA (2001) Dictionary of the Fungi. 9^th^ edn. CAB International, Wallingford.

[B21] Kornerup A, Wanscher JH (1981) Taschenlexikon der Farben. Third Edition. Muster-Schmidt Verlag, Zürich und Göttingen.

[B22] Lanfear R, Calcott B, Ho SY, Guindon S (2012) PartitionFinder: Combined selection of partitioning schemes and substitution models for phylogenetic analyses. Molecular Biology and Evolution 29: 1695–1701. 10.1093/molbev/mss020 22319168

[B23] Li X, Wang L, Wang Z (2017) Structural characterization and antioxidant activity of polysaccharide from *Hohenbuehelia serotina*. International Journal of Biological Macromolecules 98: 59–66. 10.1016/j.ijbiomac.2016.12.089 28069348

[B24] Li Y, Tolgor B, Liu Y, Li TH (2014) Flora Fungorum Sinicorum, Pleurotoid-Lentinoid Fungi. Science Press, Beijing.

[B25] Liu Y, Bau T (2009) A new species of *Hohenbuehelia* from China. Mycotaxon 108: 445–448. 10.5248/108.445

[B26] Liu Y, Bau T, Li TH (2010) Three new records of the genus *Hohenbuehelia* in China. Mycosystema 29: 454–458. 10.13346/j.mycosystema.2010.03.003

[B27] Lubian C, Kuhn OJ, Portz RL, Agustinha AM, Stangarlin JR (2021) Biological control of *Meloidogyne javanica* in bean plants by *Hohenbuehelia* spp. and *Trichoderma koningiopsis*. Arquivos do Instituto Biológico 88: e0772019. 10.1590/1808-1657000772019

[B28] Mao XL (1998) Economic fungi of Chinese. Science Press, Beijing. [In Chinese]

[B29] Mentrida CS (2016) Species delimitation and phylogenetic analyses of the genus *Hohenbuehelia* in central Europe. Dissertation, University of Wien.

[B30] Moncalvo JM, Vilgalys R, Redhead SA, Johnson JE, James TY, Aime MC, Hofstetter V, Verduin SJW, Larsson E, Baroni TJ, Thorn RG, Jacobsson S, Clémençon H, Miller OK (2002) One hundred and seventeen clades of euagarics. Molecular Phylogenetics and Evolution 23: 357–400. 10.1016/S1055-7903(02)00027-1 12099793

[B31] Phonemany M, Vadthanarat S, Raghoonundon B, Thongklang M, Raspé O (2023) Additions to *Hohenbuehelia* (*Basidiomycota*, *Pleurotaceae*): two new species and notes on *H. tristis* from northern Thailand. Mycokeys 99: 109–130. 10.3897/mycokeys.99.105317 PMC10463566 37649965

[B32] Qi LL, Ge YP, Li LY, Qin YC, Zeng H, Lang N, Li Y (2023) Two new species of *Hohenbuehelia (Pleurotaceae)* from China. Phytotaxa 578: 158–168. 10.11646/phytotaxa.578.2.2

[B33] Rambaut A, Drummond AJ, Xie D, Baele G, Suchard MA (2018) Posterior summarisation in Bayesian phylogenetics using tracer 1.7. Systematic Biology 67: 901–904. 10.1093/sysbio/syy032 PMC6101584 29718447

[B34] Rehner SA, Buckley E (2005) A *Beauveria* phylogeny inferred from nuclear ITS and EF1-alpha sequences: evidence for cryptic diversification and links to *Cordyceps* teleomorphs. Mycologia 97: 84–98. 10.3852/mycologia.97.1.84 16389960

[B35] Ronquist F, Huelsenbeck JP (2003) MrBayes 3: Bayesian phylogenetic inference under mixed models. Bioinformatics 19: 1572–1574. 10.1093/bioinformatics/btg180 12912839

[B36] Schulzer von Müggenburg S, Kanitz A, Knapp JA (1866) Die bisher bekannten Pflanzen Slavoniens. Verhandlungen der Zoologisch-Botanischen Gesellschaft in Wien 16: 1–172. 10.5962/bhl.title.9926

[B37] Shipley SM, Barr AL, Graf SJ, Collins RP, McCloud TG, Newman DJ (2006) Development of a process for the production of the anticancer lead compound pleurotin by fermentation of *Hohenbuehelia atrocaerulea*. Journal of Industrial Microbiology and Biotechnology 33: 463–468. 10.1007/s10295-006-0089-0 16501932

[B38] Silva-Filho AGS, Cortez VG (2017) *Hohenbuehelia (Pleurotaceae)* in western Paraná, Brazi. Acta Biológica Paranaense 46: 23–38. 10.5380/abpr.v46i0.54587

[B39] Singer R, Digilio APL (1951) Pródromo de la flora *Agaricina* Argentina. Lilloa 25: 6–461.

[B40] Somrithipol S, Pinruan U, Sommai S, Khamsuntorn P, Luangsa-ard JJ (2025) A new species of *Hohenbuehelia* in Nakhon Phanom, northeastern Thailand. Phytotaxa 683: 025–039. 10.11646/phytotaxa.683.1.4

[B41] Thorn RG, Barron GL (1984) Carnivorous Mushrooms. Science 224: 76–78. 10.1126/science.224.4644.76 17783527

[B42] Thorn RG, Barron GL (1986) *Nematoctonus* and the tribe *Resupinateae* in Ontario, Canada. Mycotaxon 25: 321–453. 10.5962/p.417083

[B43] Thorn RG, Moncalvo JM, Reddy CA, Vilgalys R (2000) Phylogenetic analyses and the distribution of nematophagy support a monophyletic *Pleurotaceae* within the poly phyletic pleurotoid-lentinoid fungi. Mycologia 92: 241–252. 10.1080/00275514.2000.12061151

[B44] Vilgalys R, Hester M (1990) Rapid genetic identification and mapping of enzymatically amplified ribosomal DNA from several *Cryptococcus* species. Journal of Bacteriology 172: 4238–4246. 10.1128/jb.172.8.4238-4246.1990 PMC213247 2376561

[B45] Vlasenko VA, Turmunkh D, Vlasenko AV (2022) New combination in the genus *Hohenbuehelia (Pleurotaceae)*. Phytotaxa 555: 200–204. 10.11646/phytotaxa.555.2.9

[B46] Wang L, Li X, Wang B (2019) The cytotoxicity activity of *Hohenbuehelia serotina* polyphenols on HeLa cells via induction of cell apoptosis and cell cycle arrest. Food and Chemical Toxicology 124: 239–248. 10.1016/j.fct.2018.12.001 30529121

[B47] White TJ, Bruns TD, Lee SB, Taylor JW (1990) “Amplification and direct sequencing of fungal ribosomal RNA genes for phylogenetics” in PCR Protocols: a guide to methods and applications. In: Innis MA, Gelfand DH, Sninsky JJ, White TJ (Eds). Academic Press, London. 10.1016/B978-0-12-372180-8.50042-1

[B48] Xu JZ, Jiang Y, Wang TT, Zhang D, Li XB, Hosen Md I (2023) Morphological characteristics and phylogenetic analyses revealed four new species of *Agaricales* from China. Frontiers in Microbiology 14: 1118525. 10.3389/fmicb.2023.1118525 PMC9936243 36819052

